# Placental Transfer of Conjugated Bisphenol A and Subsequent Reactivation in the Rat Fetus

**DOI:** 10.1289/ehp.0901575

**Published:** 2010-04-09

**Authors:** Miyu Nishikawa, Hidetomo Iwano, Risa Yanagisawa, Nanako Koike, Hiroki Inoue, Hiroshi Yokota

**Affiliations:** 1 Laboratory of Veterinary Biochemistry, Department of Bioscience, School of Veterinary Medicine and; 2 Department of Environmental Biochemistry, Faculty of Environmental System, Rakuno Gakuen University, Hokkaido, Japan

**Keywords:** bisphenol A, endocrine disruptors, estrogenic compounds, metabolism, transplacental

## Abstract

**Background:**

Bisphenol A (BPA), a well-known endocrine disruptor, is highly glucuronidated in the liver, and the resultant BPA-glucuronide (BPA-GA) is excreted primarily into bile. However, in rodents, prenatal exposure to low doses of BPA can adversely affect the fetus, despite the efficient drug-metabolizing systems of the dams. The transport mechanisms of BPA from mother to fetus are unknown.

**Objectives:**

To test our hypothesis that BPA-GA—an inactive metabolite—is passed through the placenta to the fetus, where it affects the fetus after reactivation, we investigated the placental transfer of BPA-GA and reactivation to BPA in the fetus.

**Methods:**

After performing uterine perfusion with BPA-GA in pregnant rats, we examined the expression and localization of the placental transporters for drug metabolites in the perfusate by reverse-transcriptase polymerase chain reaction and immunohistochemistry. We also investigated the deconjugation of BPA-GA in the fetus and examined uridine 5′-diphospho-glucuronosyltransferase (UGT) activity toward BPA and the expression of UGT isoforms in fetal liver.

**Results:**

We detected BPA-GA and deconjugated BPA in the fetus and amniotic fluid after perfusion. In the trophoblast cells, organic anion-transporting polypeptide 4a1 (Oatp4a1) was localized on the apical membrane, and multidrug resistance-associated protein 1 (Mrp1) was localized to the basolateral membrane. We observed deconjugation of BPA-GA in the fetus; furthermore, we found the expression of UGT2B1, which metabolizes BPA, to be quite low in the fetus.

**Conclusions:**

These results demonstrate that BPA-GA is transferred into the fetus and deconjugated in the fetus because of its vulnerable drug-metabolizing system.

Various chemical compounds are regarded as endocrine disruptors because they affect the homeostasis of the endocrine system, and many of these compounds are spread throughout the environment. Bisphenol A (BPA), a well-known endocrine disruptor, is polymerized to produce polycarbonate plastics and epoxy resins and is used in many industrial products. Leaching of BPA out of the products is increased by heating (Krishnan et al. 1993), contact with alkaline substances (Olea et al. 1996), and deterioration of the products (Howdeshell et al. 2003), and BPA is thus widely released into the environment. BPA has weak estrogenic activity *in vivo* and *in vitro* (Krishnan et al. 1993; Nagel et al. 1997). Several studies have demonstrated adverse effects of BPA on the reproductive (Fernández et al. 2009), nervous (MacLusky et al. 2005; Rubin et al. 2006), and immune systems (Sawai et al. 2003).

Generally, adult animals are able to metabolize and eliminate BPA from the body. Previously, we found that BPA is highly glucuronidated by UGT2B1, an isoform of uridine 5′-diphospho-glucuronosyltransferase (UGT) expressed in the rat liver (Yokota et al. 1999). In addition, our liver perfusion experiments showed that the resultant BPA-glucuronide (BPA-GA) was excreted mainly in the bile (Inoue et al. 2001). Kurebayashi et al. (2003) showed that orally administered BPA is metabolized primarily into BPA-GA and that most BPA-GA is excreted in feces via bile, although some is excreted in urine. These findings have established that BPA is almost completely eliminated by the efficient drug-metabolizing systems of adult animals (glucuronidation and excretion in the bile) during its passage through the liver. Moreover, in a reproductive toxicity study of three generations of CD Sprague-Dawley rats, Tyl et al. (2002) found no BPA-treatment–related effects at low doses (0.001–5 mg/kg/day). Again, this is most likely attributable to efficient drug metabolism of xenobiotics in an adult body.

In contrast, adverse effects of low doses of BPA exposure during pregnancy have been reported. After exposing pregnant rats to ^14^C-BPA, Domoradzki et al. (2003) detected BPA and metabolized BPA-GA in the placenta and fetus. In another study, radioactivity was detected in the fetal intestine and urinary bladder on gestational day (GD) 18 but not on GD12 or GD15 after pregnant rats were orally treated with ^14^C-BPA (Kurebayashi et al. 2005). BPA exposure during the fetal and lactational periods affects sexual differentiation of the brain structure and behavior at a dosage less than the human tolerable daily intake level (50 μg/kg; Kubo et al. 2003). In CD-1 mice, maternal exposure to 10 μg/kg BPA/day induces abnormal development of the prostate and urethra in male fetuses (Timms et al. 2005). Thus, the important issues surrounding BPA exposure involve the adverse effects not only on the generation exposed but also on the next generation, when exposure occurs during pregnancy, even at a low dose. Although a number of studies have found that BPA exposure during pregnancy induces adverse effects on the next generations, none have determined the fundamental mechanisms of these adverse effects. In particular, the mechanisms of transfer of BPA from mother to fetus are completely unknown, although this is critical to the process that affects the next generations.

In the pregnant mother and fetus, the physiological state, including the drug-metabolizing systems, differs from that of nonpregnant adult animals. Cao et al. (2002) reported that the hepatic expression of multidrug resistance-associated protein 2 (Mrp2), which excretes chemical conjugates such as glucuronide into bile duct, is reduced during pregnancy in the rat. Moreover, we previously reported that the amount of BPA-GA excreted in maternal veins is increased by compensatory excretion (Inoue et al. 2004). This suggests that the concentration of BPA-GA in maternal blood is increased for the entire gestational period and that, consequently, there is an increased risk of BPA-GA transfer across the placenta. We have also reported that in fetal rats UGT activity against xenoestrogens is absent and that this activity develops after birth, even though it is reduced in pregnant rats (Matsumoto et al. 2002). Thus, compared with the adult, the fetus has vulnerable drug-metabolizing systems, which may explain adverse effects on the fetus.

BPA-GA is a biologically inactive metabolite (Matthews et al. 2001), so BPA is considered to be safe once it has been conjugated to BPA-GA. However, during pregnancy, the concentration of BPA-GA in maternal blood may be increased because of an increase in venous excretion by hepatocytes. In the present study, we hypothesized that BPA-GA is transferred across the placenta to the fetus and then adversely affects the fetus by reactivation to BPA. To test this hypothesis, we examined whether BPA-GA is passed through the placenta and whether it is reactivated in the fetus.

## Materials and Methods

### Purification of BPA-GA after liver perfusion

BPA-GA was isolated from bile obtained by liver perfusion using our previously described method (Inoue et al. 2001). Briefly, we performed liver perfusion with BPA and collected bile. BPA-GA was then isolated from the bile by high-performance liquid chromatography (HPLC) (Shimadzu Corp., Kyoto, Japan). The isolated BPA-GA was dried in a freeze-drier (EYELA model FDU-2100; Tokyo Rikakikai Co. Ltd., Tokyo, Japan) and then dissolved in distilled water. The final concentration of BPA-GA was 650 μM.

### Animals

Pregnant Sprague-Dawley rats (GD18.5) were purchased from Sankyo Lab Co. (Tokyo, Japan). Animals were housed individually under a 12/12-hr light/dark cycle and had *ad libitum* access to water and food. For the surgical procedure and uterine perfusion, animals were treated under deep anesthesia with regard for alleviation of suffering. All experimental procedures were based on the guidelines of the Committee for Animal Welfare at Rakuno Gakuen University, which are based on the *Guide for the Care and Use of Laboratory Animals* (Institute of Laboratory Animal Resources 1996).

### Surgical procedure and uterine perfusion

**Figure 1:**
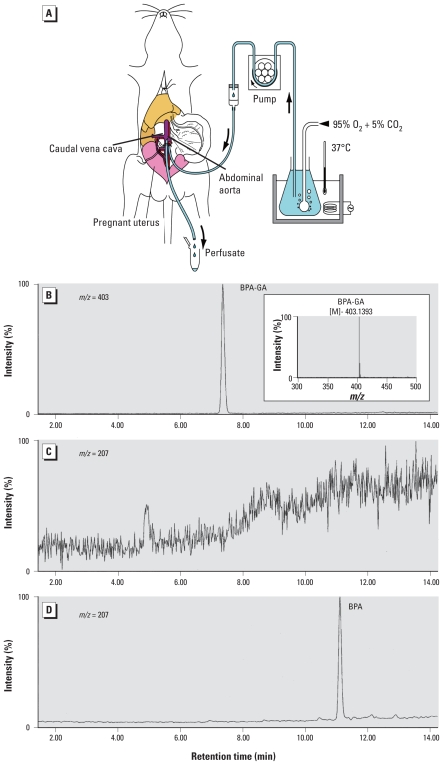
Uterine perfusion in the pregnant rat. (*A*) Schematic illustration of uterine perfusion of a pregnant rat. Perfusate with or without substrate flows into the abdominal aorta of the mother and circulates through the target placenta and fetus; the perfusate then returns to the caudal vena cava and is recovered from the drain tube. (*B–D*) Identification of BPA-GA purified from bile after liver perfusion, shown by selected ion monitoring (SIM) mass chromatograms. Using LC/TOF-MS, we confirmed the molecular mass of BPA-GA (*B*) and the absence of contamination with BPA (*C*), and we analyzed the BPA standard (*D*). (*B*) SIM mass chromatogram at *m*/*z* = 403 of the purified BPA-GA used in the present study; the inset shows the mass spectrum of purified BPA-GA. (*C*) SIM mass chromatogram (*m*/*z* = 227) of the substrate showing no contamination with BPA. (*D*) SIM mass chromatogram (*m*/*z* = 227) of the BPA standard.

A schematic illustration of uterine perfusion is shown in Figure 1A. Additional details of the surgical procedure are available in the Supplemental Material (doi:10.1289/ehp.0901575). In this perfusion system, the perfusate pumped into the abdominal aorta is circulated through one uterine artery– placenta–fetus–uterine vein unit and drips from the drain tube inserted in the caudal vein. After the perfusion, dams and fetuses were euthanized by incision of the caudal vena cava under anesthesia and tissues were collected.

We used modified Krebs-Ringer’s buffer (mKRB; 126 mM NaCl, 3 mM KCl, 1.2 mM KH_2_PO_4_, 1.3 mM MgSO_4_, 2.4 mM CaCl_2_, 10 mM glucose, 26 mM NaHCO_3_, 2.5% dextran from *Leuconostoc mesenteroides*, and 3% Dextran 70) as the perfusate. The perfusate was pumped at a constant rate of 3 mL/min. First, we perfused four animals in a preliminary group with 10 μM BPA-GA for 20 min to determine whether BPA-GA is transferred into the fetus across the placenta. In this group, we performed a preliminary perfusion with mKRB for 5 min to wash away blood, followed by 20 min inflow of mKRB containing 10 μM BPA-GA. After the perfusion, the fetus and amniotic fluid were collected.

To elucidate the kinetics of perfused BPA-GA, we used another group of four animals to compare the amount of BPA-GA in maternal veins with that in fetal tissue. After preliminary perfusion with mKRB for 5 min, pregnant rats were perfused with mKRB containing 2 μM BPA-GA for 20 min. Then, mKRB without BPA-GA was perfused for 70 min (a total of 90 min). From the beginning of the BPA-GA perfusion, the perfusate that drained from the caudal vena cava was collected from each animal at 5-min intervals for 90 min. At the end of perfusate collection, the fetus, amnion, amniotic fluid, uterus, and placenta were collected. We also performed a control study using 1-naphthol-glucuronide (1-NA-GA; Nacalai Tesque Inc., Kyoto, Japan) as the substrate. Four pregnant rats were used in each perfusion group. Values are expressed as mean ± SE.

### HPLC and liquid chromatography/time-of-flight mass spectrometry (LC/TOF-MS) analysis

Perfusates collected from the caudal vena cava were collected after uterine perfusion and prepared for HPLC and LC/TOF-MS analysis. Each perfusate sample was mixed with a 4-fold volume of acetonitrile and centrifuged at 13,000 rpm for 5 min at 4°C; each supernatant was then analyzed by HPLC.

To prepare tissues for HPLC and LC/TOF-MS analysis, we first added each sample to 0.3 mL methanol; the sample was homogenized and then sonicated for 5 min. The products were centrifuged at 13,000 rpm for 5 min at 4°C, and the supernatants were concentrated by solid-phase extraction using an Oasis HLB Plus cartridge (Waters Corp., Milford, MA, USA). The extracts were then analyzed by HPLC.

We used an HPLC system (Tosoh Corp., Tokyo, Japan) that consisted of a dual pump (DP-8020), a fluorescent photometer (FS-8020), and a column oven (CO-8020). Samples were separated at 40°C using a reverse-phase column (Unison UK-C18; Imtakt Corp., Kyoto, Japan) at a flow rate of 1.0 mL/min under a linear gradient of solution A (methanol/water = 24/76 vol/vol with 10 mM ammonium acetate) and solution B (methanol) for 20 min. BPA-GA was detected at excitation/emission of 275/308 nm and 1-NA-GA was detected at excitation/emission of 283/336 nm. The results were recorded using LC-8020 integration software (Tosoh Corp.); the elution peaks of BPA and BPA-GA were noted and the concentrations compared with the standards.

LC/TOF-MS was performed using the HPLC system described above and LCT premier XE (Waters Corp.). The flow rate of HPLC was 0.3 mL/min, and BPA-GA was monitored at *m*/*z* 403–404 by TOF-MS. In the present study, the limits of detection of BPA and BPA-GA in LC/TOF-MS were both 20 pmol/mL (data not shown).

### Antibodies

A23 anti-Mrp1 polyclonal antibody (Alexis Biochemicals) was purchased from Enzo Life Sciences Inc. (Plymouth Meeting, PA, USA). We obtained anti–organic anion-transporting polypeptide (anti-Oatp4a1) polyclonal antibody from Scrum, Inc. (Tokyo, Japan); anti-Oatp4a1 is designed to recognize the amino acid sequence LPSQSSA, which is common between human and rat (human, 705–711; rat, 703–709).

### Immunohistochemical analysis

Placental tissue samples were analyzed by immunohistochemistry, as described in detail in the Supplemental Material (doi:10.1289/ehp.0901575). Briefly, tissue sections were incubated with anti-Oatp4a1 antibody or anti-Mrp1 antibody and analyzed with a confocal laser scanning microscope (Axionvert 200M) and PASCAL software (Carl Zeiss Microimaging GmbH, Jena, Germany).

### Total RNA isolation and synthesis of cDNA

Maternal liver, placenta, fetal liver, and fetal intestine were collected on GD18.5. Total RNA was isolated from the tissues using the RNeasy Mini Kit (Qiagen, Heidelberg, Germany) according to the manufacturer’s instructions. cDNA was synthesized from total RNA using Superscript III (Invitrogen Corp., Carlsbad, CA, USA) reverse transcriptase according to the manufacturer’s instructions.

### Primers

Sequences of the oligonucleotide primers to amplify gene-specific cDNAs are available in the Supplemental Material (doi:10.1289/ehp.0901575).

### Quantitative reverse-transcription polymerase chain reaction (RT-PCR)

Quantitative expression of *Mrp1*, *Mrp2*, and *Oatp4a1* mRNA in maternal liver and placenta was investigated by real-time RT-PCR using the QuantiTect SYBR Green PCR kit (Qiagen) and analyzed by iQ5/MyiQ Single-Color (Bio-Rad Laboratories Inc., Hercules, CA, USA). Glyceraldehyde-3-phosphate dehydrogenase (*GAPDH*) was used as the internal standard. The copy number of each transporter gene was divided by that of *GAPDH* for normalization. Quantitative values are mean ± SE of four amplifications for placenta and three for maternal liver for each transporter gene.

### Incubation of fetal primary cell cultures with BPA-GA

Fetal liver and heart tissues, collected on GD18.5, were washed twice with cold phosphate-buffered saline (PBS). Tissues for two fetuses were combined in one tube and then minced in cold PBS and centrifuged for 5 min at 100 × *g* at 4°C. After removing the supernatant, the pellets were resuspended in 5 mL William’s medium (Sigma-Aldrich, Inc., St. Louis, MO, USA) containing collagenase (350 U; Wako Pure Chemical Industries, Ltd., Osaka, Japan) and DNase I (375 U; Roche Diagnostics, Basel, Switzerland) and incubated for 30 min at 37°C. After the incubation, samples were centrifuged for 5 min at 100 × g at 4°C, and the supernatants were removed. The remaining cells, which had been isolated from two fetuses, were resuspended in 200 μL William’s medium with or without 25 μM BPA-GA and incubated at 37°C for 10, 30, 60, or 120 min. At each time point, cells and medium were pooled and extracts were prepared for HPLC as described above, by adding the same volume of methanol. All experiments were repeated five times. Values express the mean ± SE.

### UGT enzyme analysis

We examined UGT enzymatic activity using hepatic microsomes. We prepared the microsomes from rat liver and performed UGT enzyme analysis as described previously by Daidoji et al (2006). See the Supplemental Material (doi:10.1289/ehp.0901575) for details.

## Results

### Passage of BPA-GA through the placenta

**Figure 2:**
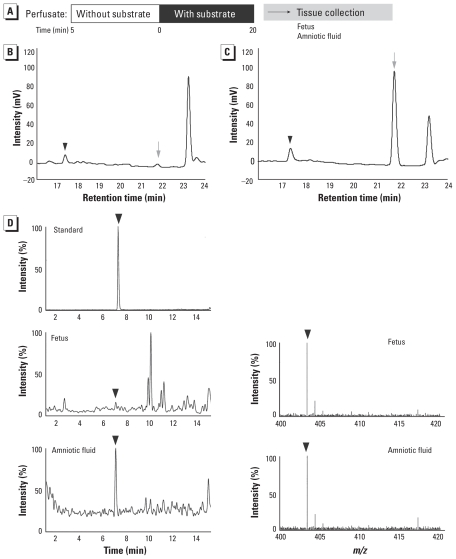
Analysis of the tissues collected after uterine perfusion. (*A*) Time schedule of the preliminary uterine perfusion with 10 μM BPA-GA for 20 min. (*B* and *C*) HPLC profiles of the extract from amniotic fluid after uterine perfusion (*B*) and standard solution containing 5 μM BPA-GA and BPA (*C*). Black arrowheads indicate BPA-GA, and gray arrows indicate BPA. (*D*) Identification of BPA-GA in standard solution (5 μM BPA-GA; top) and in the extract from the fetus and amniotic fluid after perfusion, detected by LC/TOF-MS. Left, LC chromatograms; right, mass spectra. Black arrowheads indicate BPA-GA.

First, we performed uterine perfusion (Figure 1A) using BPA-GA in pregnant rats at GD18.5 to elucidate whether BPA-GA, a major metabolite in the maternal body, can pass through the placenta into the fetus. We previously demonstrated the kinetics of various endocrine disruptors by perfusion techniques using target organs (Inoue et al. 2001; Narukawa et al. 2004). These techniques enable us to monitor the kinetics of substrates under conditions similar to the physiological state. Uterine perfusion can also mimic the original physiological state of the pregnant mother. The purity of BPA-GA isolated from perfusate after liver perfusion with BPA was confirmed by LC/TOF-MS (Figure 1B). Some studies have shown that a small amount of BPA-GA is transferred into the fetus by maternal exposure to BPA during pregnancy (Domoradzki et al. 2003). Therefore, we performed a preliminary uterine perfusion experiment (20-min inflow with 10 μM BPA-GA; Figure 2A). After the perfusion, we detected BPA-GA (Figure 2B, arrowhead) and deconjugated BPA (Figure 2B, gray arrow) in the fetus and amniotic fluid by HPLC and confirmed detection of BPA-GA by LC/TOF-MS (Figure 2D, arrowheads).

**Figure 3:**
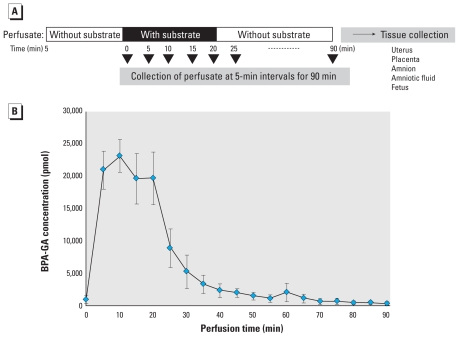
The kinetics of BPA-GA in the maternal–placental–fetal unit after uterine perfusion. (*A*) Time schedule of uterine perfusion with 2 μM BPA-GA; an additional perfusion without BPA-GA was performed for 70 min after a 20-min perfusion with BPA-GA. (*B*) Time course of concentration of BPA-GA in the collected perfusate; after 70 min, BPA-GA was barely detected in the perfusate (*n* = 4).

Next, to examine the kinetics of BPA-GA in the maternal–placental–fetal unit, we performed perfusion for 20-min inflow with a lower concentration (2 μM) of BPA-GA and additional inflow without BPA-GA for 70 min (total perfusion time, 90 min; Figure 3A). We also performed a control study using 1-NA-GA as the substrate; a total of 120 nmol substrate was used for the 20-min perfusions. After the 90-min perfusion, we detected almost all of the BPA-GA and 1-NA-GA in the perfusate collected from the maternal vein (Figure 3B and Table 1). As shown in Table 1, BPA-GA was also detected in the fetus. Moreover, deconjugated BPA was detected in amniotic fluid and in the fetus. In the fetus, we detected about 0.09% of the total amount of BPA-GA. Thus, the kinetic study detected BPA-GA and BPA in fetal tissues, although in small amounts. In contrast, we did not detect 1-NA-GA or deconjugated 1-NA except in the perfusate (Figure 3C), indicating that the placental barrier was working in this perfusion system. These data demonstrate that in this system only BPA-GA passed through the placenta into the fetus.

### Expression and localization of placental transporters

The uterine perfusion experiment showed that only BPA-GA was transferred across the placenta into the fetus. This selective transfer indicates the involvement of placental transporters. Thus, we examined the expression and localization of the placental transporters that would possibly mediate the transfer of BPA-GA. Some members of the Oatp and Mrp families are known to transport the glucuronide conjugate of steroid hormones, which structurally resemble BPA. Therefore, we focused on these two transporter families.

**Figure 4:**
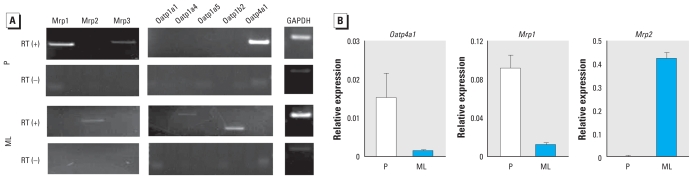
Mrp and Oatp isoforms expressed in the placenta (P) and maternal liver (ML) at GD18.5. (*A*) Expression of Mrp and Oatp isoforms examined by RT-PCR. (*B*) Quantitative analysis of *Oatp4a1*, *Mrp1*, and *Mrp2* mRNA relative to GAPDH (*n* = 4).

First, we examined the expression of the Oatp and Mrp families in the placenta and maternal liver on GD18.5 by RT-PCR. In the placenta, we detected high levels of *Mrp1* and *Oatp4a1* expression (Figure 4A). In maternal liver, we detected *Mrp2*, *Oatp1a4*, and *Oatp1b2* expression (Figure 4A). Quantitative RT-PCR confirmed the high levels of *Mrp1* and *Oatp4a1* expression in the placenta and showed that *Mrp2* was highly expressed in maternal liver (Figure 4B). These data suggest that Oatp4a1 and Mrp1 play an important role in the transport of BPA-GA from maternal blood to the fetus.

**Figure 5:**
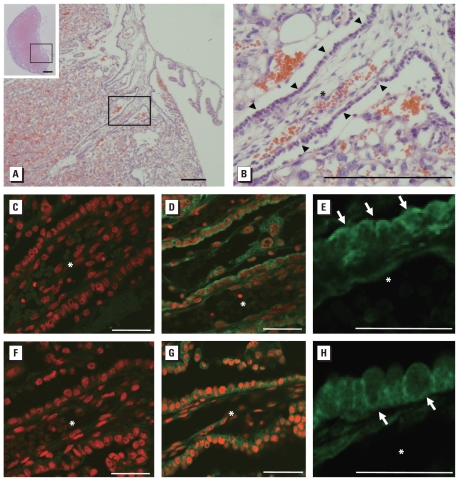
Localization of Oatp4a1 and Mrp1 in rat placenta. (*A* and *B*) Photomicrographs showing the structure of the rat placenta on GD18.5 (stained with hematoxylin and eosin). (*A*) Higher magnification of the placenta section shown in the boxed area of the inset). (*B*) Higher magnification of the boxed region in *A*; trophoblast cells (arrowheads) are localized between a fetal blood duct (asterisk) and maternal blood. In *A* and *B,* bars = 200 μm; in the inset of *A*, bar = 1 mm. (*C–E*) Immunofluorescence staining of placenta with (*D* and *E*) or without (*C*) Oatp4a1 antibody (green signal). (*F*–*H*) Immunofluorescence staining of placenta with (*G* and *H*) or without (*F*) Mrp1 antibody (green signal). In (*C–H*), nuclei are stained with propidium iodide (red); arrows indicate trophoblasts in *E* and *H*, and asterisks (*) indicate fetal blood vessels. Bars = 50 μm in *C–H*.

Mrp1 is an efflux transporter, whereas Oatp4a1 is an influx transporter. To examine the possibility of BPA-GA being transferred across the placenta by these transporters, we examined localization of Mrp1 and Oatp4a1 in the placenta by immunohistochemical analysis. The arrowheads in Figure 5B indicate trophoblast cells, which separate maternal blood from fetal blood vessels. Oatp4a1, an influx transporter, was localized on the apical membrane of the trophoblast cells (Figure 5D and arrows in Figure 5E). In contrast, Mrp1, an efflux transporter, was localized on the basolateral membrane of the trophoblast cells (Figure 5G and arrows in Figure 5H). Both Mrp1 and Oatp4a1 are known to transport endogenous estrogen conjugates, such as dehydroepiandrosterone-sulfate (DHEAS) and 17β-estradiol-glucuronide. These localization patterns suggest that BPA-GA is transferred into trophoblast cells from maternal blood vessels by Oatp4a1 and is then excreted into fetal blood from the trophoblasts by Mrp1.

### Deconjugation of BPA-GA in the fetus

**Figure 6:**
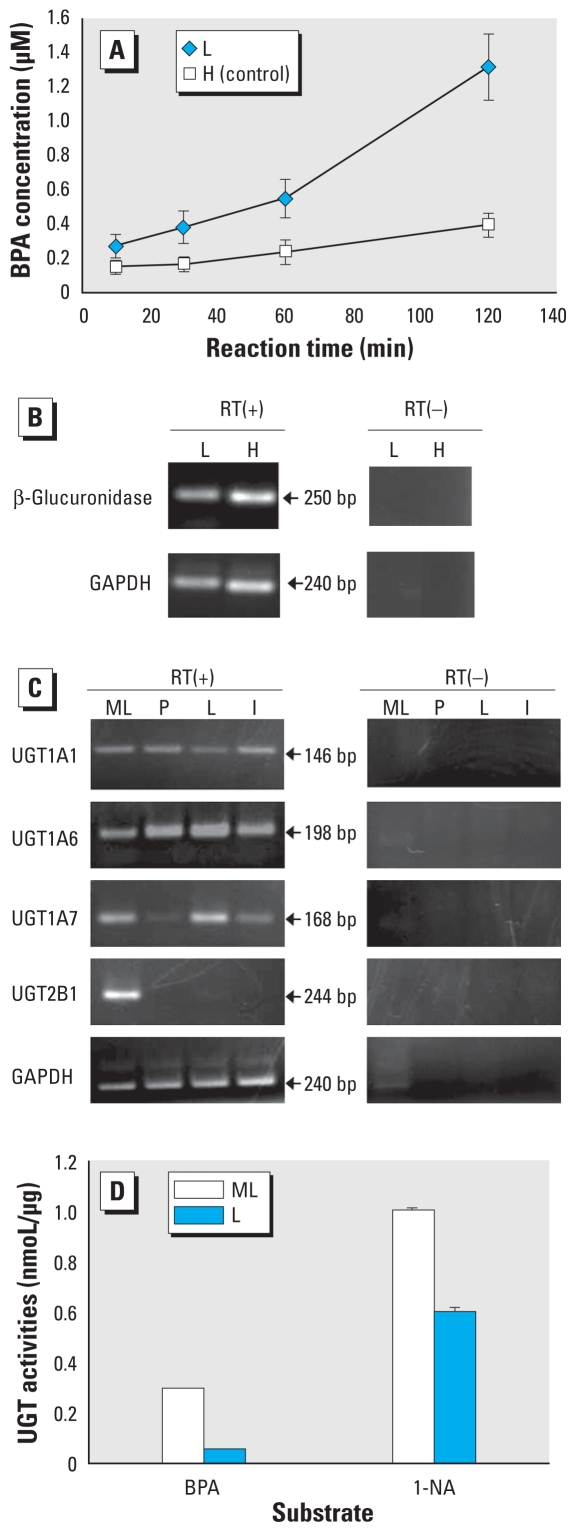
Metabolism of BPA-GA in the fetus. Abbreviations: H, fetal heart; L, fetal liver; ML, maternal liver; P, placenta. (*A*) Time course showing concentration of deconjugated BPA (mean ± SE) in the extract of cultured fetal liver and heart cells (see “Materials and Methods” for details; *n* = 5). (*B*) Expression of β*-Gase* in the fetal liver and heart on GD18.5 by RT-PCR. (*C*) Expression of UGT isoforms in ML, P, L, and I determined by RT-PCR. (*D*) UGT enzymatic activity toward BPA or 1-NA in fetal or maternal hepatic microsomes. Values shown are the concentration (mean ± SE) of glucuronide conjugates in microsome protein (nmol/μg) after 30-min reaction time (*n* = 5).

In the uterine perfusion experiment, BPA-GA passed through the placenta and was detected in the fetus. Moreover, we detected deconjugated BPA in the fetus and amniotic fluid after the perfusion (Figure 3C). These results suggest that fetal tissues have the ability to deconjugate BPA-GA. To determine the fate of BPA-GA in the fetus, we examined deconjugation of BPA-GA *in vitro*. We observed deconjugation of BPA-GA in fetal liver cells in a time-dependent manner and to a much smaller degree in fetal heart cells (Figure 6A); however, we detected the expression of β-glucuronidase (β*-Gase*), which deconjugates glucuronide conjugates, in both tissues (Figure 6B). These results indicate that BPA-GA is deconjugated to BPA in the fetus.

### Metabolism of BPA in the fetus

In a previous study (Matsumoto et al. 2002), we found that, compared with adult liver, the fetus has only a few UGT activities that glucuronidate endogenous steroid hormones and xenobiotics. This low level of UGT activity may induce delay of metabolism toward deconjugated BPA in the fetus. To examine this possibility, we determined the mRNA expression levels of the UGT isoforms in maternal liver and fetal tissue by RT-PCR. In the fetal tissues, the expression of *UGT2B1* was much lower than that in maternal liver (Figure 6C). These results are consistent with the study of Matsumoto et al. (2002). We also previously reported that UGT2B1 plays an important role in glucuronidation of BPA (Yokota et al. 1999). In contrast, other UGT isoforms examined were expressed in the fetus. UGT activities toward BPA were quite low in fetal liver microsomes compared with those in the mother, although UGT activities toward 1-NA were about 60% in maternal microsomes (Figure 6D). Taken together, the experiments in the present study indicate that the fetus has low ability to glucuronidate BPA.

## Discussion

In the present study, we found that BPA-GA passes through the placenta and is deconjugated to BPA in the fetus. To our knowledge, this is the first study to demonstrate the placental transfer of BPA-GA itself and the reactivation of BPA-GA to BPA in the fetus.

BPA-GA has long been considered to be an inactivated, safe metabolite that is eventually and inevitably excreted from the body. In addition, the placenta has been thought to act as a barrier for the fetus against xenobiotics, such as drugs and other chemical compounds. However, in the uterine perfusion experiments in the present study, the kinetic data for BPA-GA after perfusion indicate that although the placenta mostly protects the fetus from exposure to BPA-GA, a small amount of BPA-GA is in fact transferred into the fetus from maternal blood vessels via the placenta (Figure 3C). Domoradzki et al. (2003) demonstrated that the concentration of BPA-GA in the fetus is approximately 0.1% compared with that in maternal plasma after oral administration of 10 mg/kg BPA to GD16.0 rat mothers. Kurebayashi et al. (2005) also detected radioactivity in GD18 fetal tissues 24 hr after oral administration of ^14^C-BPA to the pregnant rats, but they found no radioactivity in fetuses on GD13 or GD15. The kinetics of the present study support these findings that a small amount of BPA-GA is transferred to fetus during late pregnancy.

In the present study, we detected a small amount of BPA-GA and deconjugated BPA in fetal tissues after perfusion with 2 μM BPA-GA. This perfused concentration is much higher than would be expected in a realistic situation, therefore, because the level of substrate that transfers to the fetus is low, we performed the perfusion using a high concentration of substrate for effective detection by the HPLC fluorescence photometer. The important result of the perfusion experiment is that BPA-GA was transferred across the placenta into the fetus, whereas 1-NA-GA was not. The difference in placental transfer of these substrates demonstrated that the placental function was reliable in this perfusion system. We believe that our findings suggest that humans may be exposed to BPA *in utero*, despite the fact that our experiments involved much higher exposure levels that would occur under realistic conditions. We previously reported that the excretion rate of BPA-GA from the liver into bile is decreased and that compensatory excretion of BPA-GA into the hepatic vein is increased in rodents during pregnancy (Inoue et al. 2004), and we hypothesize that this may also occur in humans. When coupled with the relatively long gestation period in humans, such compensatory increases in plasma BPA-GA could increase the risk of placental transfer of BPA-GA, even though maternal exposures may be low.

Another intriguing point is substrate specificity for placental transfer of metabolite. In the uterine perfusion with 1-NA-GA (also a type of conjugate with glucuronic acid), we did not detect 1-NA-GA or deconjugated 1-NA in the placenta or fetal tissues (Figure 3C). These results suggest that placental transfer of BPA-GA occurs in a selective manner. BPA-GA is a more hydrophilic compound than BPA, which raises the possibility that BPA-GA is transferred across the placenta in a positive manner via placental transporters. During pregnancy, nutrition and other endogenous chemical compounds are supplied to the fetus via the placenta, and metabolic waste products and xenobiotics are excreted into the maternal blood through the placenta. Thus, during pregnancy, numerous kinds of transporters that mediate the transfer of each compound are predicted to be expressed in the placenta.

Various transporters that either excrete drugs from the fetus to maternal blood or transfer nutrition and physiological compounds from the maternal body to the fetus have been isolated and characterized. Some of these transporters recognize xenobiotics because of their structural resemblance to physiological compounds. Generally, endocrine-disrupting chemicals, including BPA, often mimic endogenous chemical compounds such as estrogen. Moreover, the placenta produces steroid hormones that are used in both the mother and fetus (Strauss et al. 1996). Therefore, BPA-GA may be transferred across the placenta into the fetus by placental transporters that mediate transfer of essential endogenous physiological estrogenic compounds. Some members of the Oatp and Mrp transporter families are known to transport conjugates of steroid hormones such as DHEAS and 17β-estradiol-glucuronide, suggesting that BPA-GA is transported across the placenta by these transporters. In the present study, we observed high expression of *Oatp4a1* and *Mrp1* in the placenta by quantitative RT-PCR. High expression levels of *Oatp4a1* and *Mrp1* in rat placenta have been also reported by Leazer and Klaassen (2003). Our immunohistochemical analysis revealed that Oatp4a1 localizes on the apical membrane of trophoblast cells, and Mrp1 on the basolateral membrane. These localizations are coincident with those in humans, as reported for Oatp4a1 by Sato et al. (2003) and for Mrp1 by Nagashige et al. (2003). Oatp4a1 mediates influx transport, and Mrp1 mediates efflux transport. In view of these results, BPA-GA in maternal blood may be taken up by trophoblast by Oatp4a1 and then transferred into the fetus by Mrp1.

Some reports have indicated that cellular transfer of 1-NA-GA is mediated by the MRP family (de Vries et al. 1989; Strazielle and Ghersi-Egea 1999), but affinity of 1-NA-GA toward these transporters is still unknown. We think that the substrate specificity of Oatp4a1, which is the first trigger of influx transport, may be relevant to the selective transfer of these substrates into the fetus. Ugele et al. (2002) reported that the OATP family expressed abundantly in the human placenta (e.g., OATP-B, OATP-D, OATP-E) tended to have substrate affinity toward steroid sulfate, although OATP-E (an Oatp4a1 homolog) also had affinity toward taurocholate. These authors also demonstrated that uptake of DHEAS by monolayer trophoblast was not influenced by 1.2 mM SO_4_^2−^ in the transport buffer, indicating that SO_4_^2−^ is not an inhibitor/substrate of the steroid sulfate transporters and that the carbon backbone of the steroid sulfates is a prerequisite for inhibition/transport. The selective transport between BPA-GA and 1-NA-GA, both glucuronide conjugates, could result from the difference in affinity not only for the conjugate residue but also for the parent residue. Further study is required to confirm the affinity of BPA-GA to these transporters. Placenta-specific expression of *Oatp4a1* has already been reported (Cheng et al. 2005; Leazer and Klaassen 2003); however, the physiological role of Oatp4a1 in the placenta is still unclear. Analysis of the functions of Oatp4a1 will lead to greater understanding of this molecule’s placental functions regarding the synthesis, metabolism, and kinetics of hormones.

In the present study, we confirmed the purity of BPA-GA obtained from bile after liver perfusion using LC/TOF-MS (Figure 1B). Nevertheless, we detected not only BPA-GA but also deconjugated BPA in the fetus and amniotic fluid after uterine perfusion. The detection of BPA indicates the possibility that BPA-GA is deconjugated by fetal β-Gase. Certain organs, such as the lung, small intestine, and placenta, have high β-Gase activity, which causes release of glucuronic acid from a glucuronide conjugate (Paigen 1989; Sperker et al. 1997). Thus, we examined the possibility of deconjugation of BPA-GA in fetal tissue.

After co-incubation with BPA-GA, we detected deconjugated BPA in fetal liver and to a small degree in fetal heart (Figure 6A). We observed β*-Gase* expression in both tissues (Figure 6B). Interestingly, St-Pierre et al. (2004) reported that the expression of *Oatp4a1* in fetal liver is approximately 10^5^ times higher than that in adult liver, although this expression level is still very low compared with placenta. The difference in deconjugation rates among tissues in the present study may reflect uptake into cells via transporters rather than through β-Gase expression. Another possibility is that BPA-GA is deconjugated in the placenta and then resultant BPA is transferred into the fetus by passive diffusion. Therefore, we also examined deconjugation of BPA-GA in placental trophoblast primary cell culture. We did not observe deconjugation in the trophoblast cells, but we did observe β*-Gase* expression (data not shown). The possibility of transfer of placental deconjugated BPA could not be excluded completely in this experiment. Further work is required to examine the tissue-specific deconjugation of BPA-GA and the possibility of BPA transfer. At any rate, these data demonstrate that once BPA-GA is transferred into the fetus, it is deconjugated (reactivated) to BPA, and that this may be due to uptake of BPA-GA into fetal cells by transporters and subsequent catalysis by β-Gase.

When contemplating whether deconjugated BPA adversely affects the fetus, it is most important to consider the fetal drug- metabolizing system, especially in relation to UGT2B1 activity, which glucuronidates BPA to BPA-GA (Yokota et al. 1999). UGT isoforms glucuronidate endogenous physiological compounds and other xenobiotics (Webb et al. 2005). We previously reported that UGT activity in the fetus is quite low, compared with that in the adult, and develops gradually after birth (Matsumoto et al. 2002). In the present study, the expression of *UGT2B1* mRNA in fetal organs was much lower than that in maternal liver. Moreover, in the UGT activity assay using liver microsomes on GD18.5, UGT activities in the fetal liver microsomes toward BPA were quite low. In this experiment, the ratio of fetal UGT activity toward BPA was about 20% of maternal microsomes (Figure 6D). However, we also previously showed that the UGT activity toward BPA in pregnant rats on GD19.0 were decreased to about 70% of that of nonpregnant adult rats (Matsumoto et al. 2002). Therefore, in theory, the fetal UGT activity toward BPA during late pregnancy may be < 15% of that in nonpregnant adult rats. On the other hand, the fetal UGT activity toward 1-NA was higher than that toward BPA, and we observed abundant expression of *UGT1A6*, which is known to glucuronidate 1-NA, in fetal tissues. Although we observed the expression of other UGT isoforms, such as *UGT1A1*, *UGT1A6*, and *UGT1A7*, in the fetus (Figure 6C), the results of the UGT activity assay strongly suggested that the fetus has low ability to metabolize BPA due to a deficiency in UGT2B1. UGT2B1 has also been shown to glucuronidate some xenobiotics such as 4-hydroxybiphenyl and opioid compounds (King et al. 1997). This suggests that there are different risks to the fetus according to the xenobiotic involved. In particular, exposure to chemical compounds that are glucuronidated by UGT2B1 is critical for the fetus.

**Figure 7:**
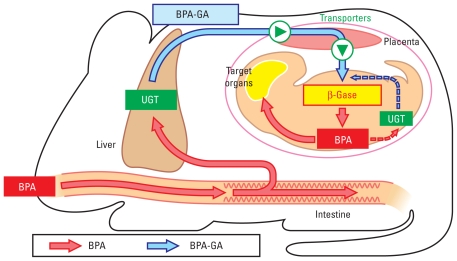
The predicted mechanism of adverse effects on the fetus induced by maternal BPA exposure during pregnancy. BPA-GA in the maternal blood is transferred across the placenta to the fetus and then deconjugated to BPA. Deconjugated BPA may remain in the fetus because of a deficiency in fetal UGT activities.

A series of results in the present study suggest that the fate of some glucuronide-conjugated chemical compounds in the maternal body may be similar to that of BPA-GA during pregnancy. Once the glucuronide-conjugate is transferred into the fetus and deconjugated, it may be difficult to protect the fetus from exposure to reactivated chemicals because of its vulnerable drug-metabolizing system (Figure 7). The glucuronide conjugate was previously considered to be inactive and therefore nontoxic. Therefore, when we assess the toxicity of xenobiotics, it is important to consider the placental transfer of inactive metabolites such as BPA-GA.

Rodent placenta, including rat, is used as a model of human placenta because of its structural similarities (placenta hemochorialis). There are, however, some important differences between rodent and human placental structure. In rodents, maternal blood and fetal blood vessels are primarily separated by two syncytiotrophoblast layers, but in humans, only a syncytiotrophoblast monolayer separates fetal blood vessels from maternal blood (Malassiné et al. 2003). Therefore, it is possible that the human fetus is more sensitive and at greater risk than is the rodent fetus.

In human liver microsomes, UGT2B15 is a major isoform that glucuronidates BPA (Hanioka et al. 2008). Moreover, Strassburg et al. (2002) observed that in human fetal liver at 20 weeks gestation, *UGT2B15* was not expressed as well as other UGT isoforms examined. Both of these studies also suggest that human fetuses also have metabolizing systems vulnerable to BPA.

## Conclusion

Our study had three major findings. First, BPA-GA is transferred into the fetus through the placenta, even if only in small amounts. Second, BPA-GA is deconjugated to BPA in the fetus. Third, fetal liver microsomes have low ability to metabolize BPA to BPA-GA, as supported by the low expression of *UGT2B1* in the fetus. BPA may have been detected in the fetus after the perfusion due to high deconjugation ability of BPA-GA and low UGT activity toward BPA. Thus, we hypothesize that prenatal exposure to BPA may occur at levels sufficient to influence fetal development because of the exceptional drug-metabolizing system of the mother and fetus, which is particular to pregnancy. In 2003, 6 billion pounds of BPA were produced worldwide (Burridge 2003). Furthermore, BPA has been shown to be easily released from products. Therefore, it is impossible to completely protect pregnant women from exposure to BPA. However, understanding the mechanism by which BPA-GA transfers across the placenta may allow protection of the fetus from exposure to BPA and its metabolites. Further study is necessary to elucidate the process of BPA-GA transfer across the placenta.

## Figures and Tables

**Table 1:** Distribution of the substrate after perfusion, shown as the amount (mean ± SD) of substrate detected in each sample.

	Substrate			
	BPA-GA		1-NA-GA	
Tissues	BPA-GA	BPA	1-NA-GA	1NA
Perfusate (maternal side)	113.10 ± 6.92 nmol	ND	111.77 ± 2.58 nmol	ND
Uterus	ND	ND	ND	ND
Placenta	ND	ND	ND	ND
Amnion	ND	ND	ND	ND
Amniotic fluid	ND	31.35 ± 4.52 pmol	ND	ND
Fetus	109.26 ± 36.75 pmol	6.45 ± 1.45 pmol	ND	ND

ND, not detected. The total amount of substrate detected in sample was calculated from the area under the curve shown in Figure 3B. *n* = 4 for each tissue type per treatment group.
